# Parenting with a History of ACEs: Examining the Impact of Cumulative Exposure and Child Sexual Abuse on Mothers’ and Fathers’ Reports of Parent-Child Relationship Quality

**DOI:** 10.1007/s40653-026-00895-2

**Published:** 2026-04-29

**Authors:** Vanesa Pastor-Cerezo, Nicole Maiorano, Lorraine Swords

**Affiliations:** 1 University of Alcalá, Alcalá de Henares, Spain; 2 Trinity College Dublin, Dublin, Ireland

**Keywords:** Adverse childhood experiences, Child sexual abuse, Parent–child relationship, Closeness, Conflict

## Abstract

This study investigates whether cumulative Adverse Childhood Experiences (ACEs) and specific exposure to child sexual abuse (CSA) are associated with parent reports of closeness and conflict in their relationships with their children. Parents (*N* = 426; 85.9% mothers and 14.01% fathers) were recruited from family support services in Ireland and invited to complete a survey that included an assessment of their exposure to abuse, neglect and household disfunction during their childhood or adolescence. In addition, they completed a measure of their perceived relationship quality with their children. Data were analysed using hierarchal regression and found that parents’ greater cumulative ACE scores were associated with less closeness and greater conflict in parent-child relationships. CSA exposure was significantly associated with conflict in bivariate analysis, but this association was no longer significant once sociodemographic and cumulative ACEs were accounted for in regression. Findings provide insight into the intergenerational impact of childhood adversity and are discussed in terms of informing the design of trauma-informed family support interventions.

## Introduction

Since the seminal work of Felitti and colleagues (1998), the study of adverse childhood experiences (ACEs), and their long-term correlates, has grown substantially. ACE research has demonstrated that exposure to potentially traumatic experiences in childhood is not uncommon, with approximately 60% of adults globally reporting that they had experienced some form of abuse, neglect or household dysfunction (e.g. domestic violence, incarcerated family members) before the age of 18 years (Madigan et al., 2023; Swedo et al., 2023). Researchers have also learned that individuals who experience one ACE are more likely to experience another (e.g. Lacey et al., 2020), and that there is a cumulative, negative effect of ACEs on health, whereby a tally of four or more ACEs leaves those affected at an increased risk of a range of health conditions such as heart disease, cancer, respiratory disease and mental illness (Hughes et al., 2017).

More recently studies demonstrating a correlation between parent and child ACE scores have called into consideration the potential intergeneration effects of ACEs (Schickedanz et al., 2021). Multiple pathways have been proposed to explain this transmission. For example, substance use or mental illness in adulthood that results from early adversity experiences represent ACE risks for the next generation (e.g. Swords et al., 2024; Zhang et al., 2023). Additionally, parents with childhoods featuring maltreatment may be less able to reference examples of warm and responsive caregiving to guide their relational approach with their own children (e.g. Narayan et al., 2021; Sroufe, 2017). This theory was supported by a systematic review of ACE literature that found an association between parental ACEs and decreased emotional availability and increased permissive and authoritarian discipline strategies through attachment theory (Rowell & Neal-Barnett, 2021).

Much of the ACE literature thus far considers ACEs as a cumulative score, whereby a value of one is assigned for each adversity experienced at any time throughout childhood, and these experiences are summed. While cumulative ACE scores have been instrumental in understanding the impact of potentially traumatic events in early life, they also assume that each adversity counted as ‘one’ is equally important and impactful, and neglect specific patterning of ACEs (Lacey & Minnis, 2019). Cumulative indices do not consider the timing, duration and or intensity of the adversity experience, their multiplicative and conditional nature, or their differential impact on a child’s later life. Consequently, a cumulative ACE score cannot disentangle how some adversity experiences may be related more strongly to particular outcomes than others.

One type of adversity that has received considerable research attention is child sexual abuse (CSA). CSA has been defined by ECPAT International (2016) as the search for sexual gratification through contact or non-contact acts with minors. It is pervasive, affecting an estimated one in five girls and one in seven boys globally (UNICEF, 2023). Extensive evidence spanning psychological, neurobiological, and epidemiological research indicates that it is one of the most severe interpersonal childhood traumas causing profound, long-lasting damage to mental health, physical well-being, and the ability to form relationships (Pastor-Cerezo & Iborra Cuéllar 2026a, 2026b). Large bodies of research have shown that a history of experiencing CSA is associated with a broad array of risk behaviours and poorer health outcomes in adulthood, including alcohol and substance use, suicide attempts, and mental disorders such as depression and anxiety (e.g. Amado et al., 2015; Fergusson et al., 2013; Irish et al., 2010). For some specific outcomes, for example, exposure to sexual victimization in adulthood, CSA is the single largest risk factor among all ACE types (Ports et al., 2016).

Due to its association with specific traumatic reactions including sexualized behaviours and severe trust issues, the experience of CSA also uniquely affects interpersonal relationships. In the short-term, CSA severely undermines the child’s sense of safety that is central to secure attachments (Finkelhor & Browne, 1985). Further, when committed by a caregiver or family member, CSA turns what should be a source of comfort into a source of harm. This betrayal leads to heightened conflict within the family, as the child’s emotional distress and behavioural reactions to the abuse often increase tension and misunderstanding (Deblinger & Heflin, 1996). Pastor-Cerezo & Iborra Cuéllar (2026b) found that the relational effects of sexual abuse in childhood carry through to adulthood causing difficulties in intimate relationships that are not limited to sexual activity. The authors propose that affected individuals develop a fearful adult attachment style from having a negative working model of themselves and others. They have difficulties with emotional bonding and ultimately withdraw from relationships, despite the need for attention and closeness (Pastor-Cerezo & Iborra Cuéllar 2026a).

Similarly to other ACEs, the long-term, relational effects of CSA may affect the parenting style of survivors. Research has proposed multiple effects of CSA on parenting behaviours including promoting role reversal (e.g. parents being overly emotionally dependent on their child) and permissive parenting (i.e., the lack of appropriate boundaries and discipline, e.g. Alexander et al., 2000). Experiencing CSA also decreases confidence in parenting (DiLillo & Damashek, 2003). The unique effect of CSA, though, may not be adequately captured by this literature. In a review of CSA’s effect on parenting, Hugill et al. (2017) note the lack of consideration of other ACEs within analyses. Given the high co-occurrence of ACEs (Felitti et al., 1998), and specifically of CSA to other ACEs (Easton, 2012), a failure to account for other ACEs prevents an understanding of the specific effect of CSA as results may be attributed to other unmeasured traumatic experiences. A study by Barrett (2009) did control for other forms of adversity, finding that the relationship between CSA and parental warmth and aggression was no longer significant after including sociodemographic factors and other forms of childhood adversity. However, this study included a sample of predominately African-American mothers from America, potentially limiting its generalisability to other populations, and reflecting the research field’s focus on mothers (e.g., DiLillo & Damashek, 2003).

The present study aims to further examine the relationship between parents’ reports of early adversity and their perception of their parent-child relationship, within an Irish sample. Specifically, parents in this study were recruited from therapeutic family support services they attended. Self-referral for these services is accepted, but typically families are referred from other sources like schools, their family doctor, or the courts system. The service provides a wide range of needs-led, solution-focussed supports that include individual and family work, parenting programmes, and home visits. Madigan et al. (2023) noted that although childhood adversity experiences are not uncommon in the general population, members of some subgroups, such as those experiencing economic difficulties or those with a history of addiction or mental health issues, are at heightened risk. As such, they propose that research focusing on ACE disparities within subgroups of the population should be a priority for public health. In addition, from a family services perspective, in order for interventions to be targeted, culturally-responsive and, importantly, effective, understanding local level presentations of risk and resilience is key.

Utilising an adapted 11-item ACE scale developed by Felitti and colleagues (1998) along with the Child-Parent Relationship Scale (CPRS) developed by Pianta (1992), the present study will analyse relationships between cumulative ACEs generally, and CSA specifically, and parents’ report of closeness and conflict with their child. It seeks to uncover what effect exposure to adversity in parents’ own childhood may have on their reports of closeness and conflict in their relationships with their children. The following hypotheses will be tested: (1) Greater ACE exposure will predict less closeness and more conflict in parent-child relationships, and (2) CSA as a specific ACE will predict less closeness and more conflict in parent-child relationships after cumulative ACE exposure is accounted for.

## Method

This study is part of a broader research programme funded by an Irish Non-Governmental Organisation (NGO) to evaluate the effectiveness of their therapeutic family support services (see Spratt et al., 2021; Monson et al., 2022). Ethical approval was granted by [*redacted for peer review*].

### Participants

Four hundred and twenty-six parents attending community-based therapeutic family services with their children participated in this study. The majority of parents (85.9%, *n* = 366) were mothers (age range 21 to 59 years, mean age 38.8), with fathers (age range 19 to 56 years, mean age 38.9) making up 14.01% (*n* = 60) of the sample. Over three quarters of parents were Irish (78.4%) with the rest reporting a range of nationalities from across Europe, Asia and Africa. While 6.1% of parents had not progressed beyond primary level education, and 19.6% had not completed second level education, 22.5% of parents had completed their formal second level examinations and just over half of the sample had received a third level certificate or diploma (31.2%), or a third level primary (10.6%) or higher (9.9%) degree.

With the support of a family caseworker, parents were invited to complete a survey measuring various aspects of parent and child wellbeing, health and relationship quality. They were asked to respond to the questions while keeping in mind their child who was presently of greatest concern to them. There was a more equal gender balance among the children that parents were responding in relation to, with 55.6% of parents (*n* = 237) reporting on their sons (5 months to 18 years; mean age 9.7 years) and 44.4% of parents (3 months to 18 years; *n* = 189) reporting on their daughters (mean age 10.9 years).

### Materials

Using standardised measures and individual socio-demographic questions, the survey instrument captured a range of information relating to the child and parent. The socio-demographic data collected comprised child and parent age and sex. Parents’ highest level of education was chosen as a proxy for family socio-economic status (SES) as it is widely used as a reliable indicator of SES in cross-sectional and longitudinal studies (e.g. Calatrava et al., 2023; Nixon et al., 2013; Nixon & Swords, 2016) because it represents a stable resource that influences opportunities and outcomes across generations. Additionally, it correlates with other measures of socioeconomic status, like household material conditions and financial income or strain.

The measures relevant to the present study are as follows:

- **Adverse Childhood Experiences (ACEs)**
  This was measured using an 11-item scale that tallies exposure to a range of potentially traumatising life situations or events prior to an individual’s 18th birthday. It was adapted from the original ten-item ACE measure developed by Felitti et al. (1998). This original measure contained five items assessing abuse (e.g., emotional, sexual) and neglect directed against the individual, and five relating to difficult family circumstances (e.g., domestic abuse, having a parent in prison). The additional item in the present study asks about domestic abuse directed at fathers (the original list only asked about domestic abuse directed at mothers). Respondents’ final score can thus range from zero (no adverse early experiences) to eleven (all items from the scale has been experienced).
- **Parent-Child Relationship Quality**
  Closeness and conflict in the parent-child relationship was assessed using the Pianta Child-Parent Relationship Scale (Pianta, 1992), a self-report measure completed by parents to assess the quality of their relationship with the child. The measure consists of 15 items rated on a five-point Likert scale. The seven-item Closeness subscale includes statements such as “I share an affectionate, warm relationship with my child”, while the eight-item Conflict subscale poses statements like, “When my child is in a bad mood, I know we’re in for a long and difficult day”. All items are rated on a five-point scale from ‘definitely does not apply’ to ‘definitely applies’ so that scores on the Closeness subscale can range from 7 (low levels of closeness) to 35 (high levels of closeness) and scores on the Conflict subscale can range from 8 (low levels of conflict) to 40 (high levels of conflict). Cronbach’s alpha in the present study was acceptable both for Closeness (α =.74) and for Conflict (α =.85).

### Procedure

Parents were recruited between 2017 and 2020 upon uptake of therapeutic family services in the eastern region of Ireland. There were no exclusion criteria. Parents were given information as to the purposes of the study and invited to complete a survey with the support of a family caseworker. They were assured that participation was entirely voluntary and would in no way affect their access to services. They were further assured that, should they agree to take part, their responses would be fully anonymised before being transferred to the research team for analysis. Those who agreed to take part signed an informed consent document and were provided with the survey. For questions relating to their children (for example, questions about conflict and closeness in the parent-child relationship), if they had more than one, they were asked to keep in mind the child they were most concerned about and attending services in relation to. The survey took approximately twenty minutes to complete. Parents were thanked for their participation and were reminded of supports available to them within the service should they wish to discuss any issues raised during the course of their participation.

### Analysis Plan

The data for the present study are comprised of responses provided by both mothers and fathers on measures of early adversity and the parenting domains of closeness and conflict. Initial descriptive analyses gauged the percentage of parents reporting different quantities and types of Adverse Childhood Experiences. Chi-square analyses were then conducted to determine if parents with childhood experiences of sexual abuse (CSA) were more likely to experience other forms of childhood adversity than parents who had experienced ACES, but not specifically CSA. Bivariate analyses were conducted to assess the relationship between adversity and parent-child closeness and conflict. Hierarchical multiple regression was then used to determine if any significant bivariate relationships remained statistically significant after controlling for the sociodemographic variables of child age and sex and parent sex and level of education.

## Results

### Parents’ ACE Exposure

**Fig. 1:**
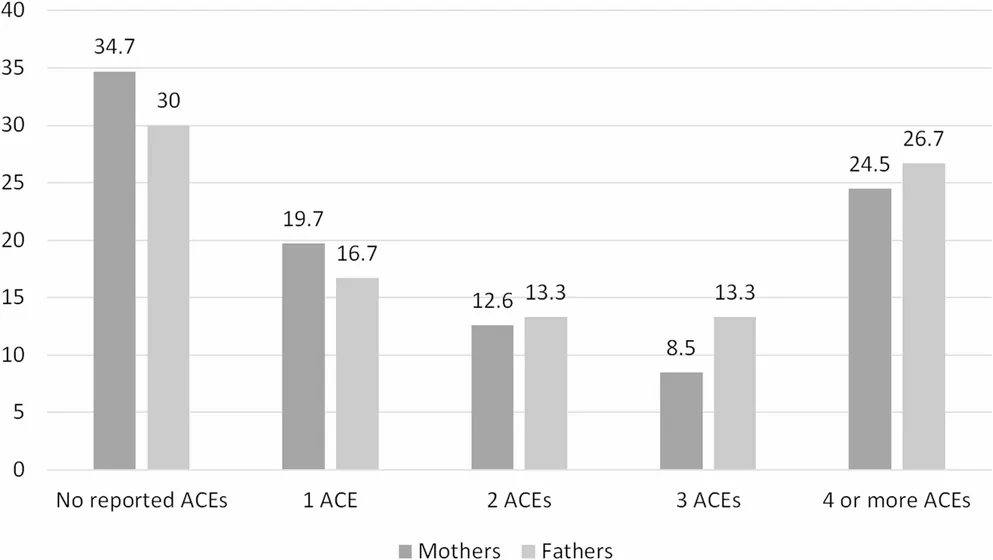
Percentage of mothers and fathers reporting different quantities of adverse childhood experiences

The figure below (Fig. 1) illustrates the number of adverse life events that mothers and fathers reported experiencing as children or adolescents. For mothers, 65.3% encountered at least one and the average number of ACEs experienced by this group was 3.26 (range: One ACE to 11 ACEs). For fathers, 70% encountered at least one and the average number of ACEs experienced by this group was 3.6 (range: one ACE to nine ACEs).

Childhood sexual abuse was reported by 10.4% of mothers and 3.3% of fathers. Due to the proportionately smaller number of fathers reporting CSA, data from mothers and fathers were combined to determine if parents with childhood experiences of sexual abuse (CSA) were more likely to experience other forms of childhood adversity than parents who had experienced ACES, but not specifically CSA. Results indicated that parents reporting CSA were significantly more likely to also experience the following adversities in their childhood: psychological abuse (χ^2^ (1, *N* = 281) = 12.46, *p* <.001), physical abuse (χ^2^ (1, *N* = 279) = 11.43, *p* <.001), emotional neglect (χ^2^ (1, *N* = 278) = 23.98, *p* <.001), physical neglect (χ^2^ (1, *N* = 281) = 8.33, *p* <.05), and domestic abuse [with regard to mothers being abused: χ^2^ (1, *N* = 281) = 6.98, *p* <.05; with regard to fathers being abused: χ^2^ (1, *N* = 275) = 13.32, *p* <.001]. Among parents with experience of at least one adversity in childhood, those reporting CSA had a significantly higher mean cumulative ACE score (5.8) in comparison with parents reporting adversity experiences but without CSA (2.9), (*t* (279) = 7.82, *p* <.001).

### The Effect of ACE Exposure on Parent-Child Relationships

First, descriptive and correlation analyses were performed (Table 1), for which mothers’ and fathers’ responses were combined for analysis. Correlation analyses indicate that increases in ACE total scores for parents are associated with small but significant increases in reports of conflict and small but significant decreases in reports of closeness. Parents’ experiences of CSA were weakly, but significantly, associated with more conflict, but not significantly less closeness. Moreover, the sample mean scores of 30.31 for parent-child relationship closeness is at the higher end of the scale (range 7–35), indicating that parents generally report good quality relationship interactions with their children. For context, comparison with data from a nationally representative sample of parents in Ireland reporting on their relationships with their 13-year-olds indicates that scores for closeness are similar, averaging at 30.5 for fathers and 32.95 for mothers (Nixon, 2021). However, the mean conflict score of 22.69 in the present study is higher than the average conflict scores reported in Nixon (2021) at 15.1 for fathers and 15.3 for mothers. This suggests that arguments and discord are not an uncommon feature of many of the families’ interactions.

**Table 1:** Descriptive data and bivariate Pearson’s correlations for key variables

	Min/Max	M (SD)	PT-ACE	PE-CSA	PC-Cl	PC-Co
PT-ACE	0/11	2.18 (2.49)	1	0.375**	−0.117**	0.109*
PE-CSA	No/Yes	N/A	0.375**	1	−0.048	0.087*
PC-Cl	12/35	30.31 (4.58)	−0.117**	−0.019	1	−0.477**
PC-Co	8/40	2.18 (2.49)	1	0.375**	−0.117**	0.109*

*PT-ACE *Parents’ Total ACEs, *PE-CSA *Parents’ Experience of CSA, *PC-Cl *Parent-Child Closeness, *PC-Co *Parent-Child Conflict

Statistical note. Correlation is significant at the 0.01 level (one-tailed) **; Correlation is significant at the 0.05 level (one-tailed) *

A hierarchical regression analysis was conducted to examine whether parents’ reports of closeness with their children were associated with their cumulative and specific ACE exposure (Table 2). In Step 1, child sex and age, parent sex, and parent education were entered as covariates. This initial model was significant (F (4,422) = 10.27, *p* <.001) and accounted for approximately 9% of the variance in parent–child closeness. In this step, both child sex and child age were significant predictors of closeness, whereas parent sex and parent education were not. In Step 2, total ACE count was added. This model remained significant (F (5,421) = 9.57, *p* <.001) and explained about 10% of the variance. The inclusion of total ACEs led to a significant improvement in model fit compared to Step 1 (ΔF (1,421) = 6.25, *p* =.013, ΔR^2^ = 0.01). Higher cumulative ACE exposure was associated with lower perceived closeness. In Step 3, experience of childhood sexual abuse was entered. The final model was still significant overall (F (6,420) = 7.96, *p* <.001) but adding CSA did not improve prediction beyond total ACEs and the covariates (ΔF (1,420) = 0.03, *p* =.983, ΔR^2^ ≈ 0.00). CSA was not a unique predictor of closeness once other variables were included. Total ACE exposure, however, continued to show a negative association with parent–child closeness in the final model.

**Table 2:** Coefficients for variables predicting parent-child closeness

			At entry to model				Final model			
Variable	R^2^	ΔR^2^	B	SE B	β	t	B	SE B	β	t
Step 1	0.09***									
Child sexᵃ			1.51	0.44	0.16***	3.48	1.46	0.43	0.16***	3.37
Child age			− 0.31	0.06	− 0.27***	−5.67	− 0.32	0.06	− 0.27***	−5.77
Parent sexᵃ			0.48	0.62	0.04	0.78	0.42	0.62	0.03	0.68
Parent education			0.15	0.16	0.04	0.93	0.08	0.16	0.02	0.51
Step 2	0.10***	0.01*								
Total ACE count			− 0.22	0.09	− 0.12*	−2.50	− 0.22	0.10	− 0.12*	−2.19
Step 3	0.10***	0.00								
CSAᵇ			− 0.02	0.83	0.00	−0.02	− 0.02	0.83	0.00	−0.02

*ACE *Adverse Childhood Experiences, *CSA *childhood sexual abuse. / aChild Sex and Parent Sex coded as 0 = male, 1 = female; bCSA coded as 0 = no, 1 = yes

Statistical note. Higher Total ACE Count score indicates greater cumulative exposure to adverse childhood experiences

p < .05. ** p < .01. *** p < .001

A second hierarchical regression analysis was conducted to examine if parents’ reports of conflict in their relationship with their children was predicted by their cumulative and specific ACE exposure (Table 3). As before, the sociodemographic variables of child and parent sex, child age, and parent highest level of education were first added to the model. This initial model was significant (F (4,422) = 7.03, *p* <.001) accounting for approximately 6% of the variance in parent–child conflict. In this step, child sex, child age, and parent sex emerged as significant predictors, whereas parent education was not. In Step 2, total ACE count was added to the model. The model remained significant (F (5,421) = 6.99, *p* <.001) explaining about 8% of the variance in conflict. The inclusion of total ACEs significantly improved model fit compared to Step 1 (ΔF (1,421) = 6.43, *p* =.012, ΔR^2^ = 0.01). Higher cumulative ACE exposure was associated with higher reported levels of parent–child conflict. In Step 3, experience of childhood sexual abuse was entered. The final model was significant overall (F (6,420) = 5.84, *p* <.001) but the addition of CSA did not improve prediction beyond total ACEs and the covariates (ΔF (1,420) = 0.48, *p* =.63, ΔR^2^ ≈ 0.00). CSA was not a unique predictor of conflict once other variables were included.

**Table 3:** Coefficients for variables predicting parent-child conflict

			At entry to model				Final model			
Variable	*R*²	ΔR²	B	SE B	β	t	B	SE B	β	t
Step 1	0.06***									
Child sexᵃ			−2.07	0.84	− 0.12*	−2.47	−1.99	0.84	− 0.11*	−2.38
Child age			0.36	0.11	0.16***	3.33	0.37	0.11	0.17***	3.44
Parent sexᵃ			3.74	1.19	0.15**	3.14	3.79	1.19	0.15**	3.18
Parent education			− 0.28	0.31	− 0.04	− 0.89	− 0.15	0.31	− 0.02	− 0.48
Step 2	0.08***	0.01*								
Total ACE count			0.43	0.17	0.12*	2.53	0.38	0.19	0.11*	1.99
Step 3	0.08***	0.00								
CSAᵇ			0.77	1.60	0.03	0.48	0.77	1.60	0.03	0.48

*ACE *Adverse Childhood Experiences, *CSA *childhood sexual abuse./^a^Child Sex and Parent Sex coded as 0 = male, 1 = female; ^b^CSA coded as 0 = no, 1 = yes

Statistical note. Higher Total ACE Count score indicates greater cumulative exposure to adverse childhood experiences

p <.05. ** p <.01. *** p <.001

## Discussion

The present study sought to describe the ACE histories of a sample of mothers and fathers attending family support services in Ireland and examine the relationship between adversities in their own childhood and their reports of closeness and conflict in their relationships with their children. Findings indicated that the majority of parents had experienced at least one adversity and those reporting CSA had a significantly higher mean cumulative ACE score than parents reporting adversity experiences that did not involve CSA. Cumulative ACE scores were significantly associated with less closeness and more conflict. CSA was also associated with more conflict, but no relationship was found between CSA and closeness. Following analyses that controlled for both child and parent sociodemographic factors, cumulative ACE scores were noted to still significantly predict both parenting outcomes. The statistically significant impact of CSA on levels of parent-child conflict noted in bivariate analyses was not significant after controlling for sociodemographic variables and cumulative childhood adversity in the regression equation.

These findings indicate the importance of understanding and accounting for the cumulative nature of ACEs. Specifically, the doubled average cumulative ACE score for parents who experienced CSA compared to parents who experienced other adversities outside of CSA requires consideration for research that aims to understand a potential unique impact of CSA. Previous research has proposed that CSA uniquely impacts on parenting by affecting parenting behaviours such as boundary setting and discipling (Alexander et al., 2000; MacIntosh & Ménard, 2021). However, the current finding indicates the need to consider that in testing the relationship between CSA and parenting outcomes, the impact of other childhood adversities may also be captured. Previous research examining parenting outcomes affected by CSA may not be accounting for these overlapping experiences (Greene et al., 2020). Similarly, service-providers for survivors of CSA should consider the likelihood of the survivor experiencing multiple other ACEs that can affect their mental health and wellbeing (Hughes et al., 2017). The authors pose that rather than attempting to “disentangle” CSA from ACEs, the current results highlight the need to understand ACEs’ compounding and interacting nature. However, future research should utilise additional analytical approaches to explore this in more depth. For example, latent class analysis has been increasingly used to analyse potential ACE typologies and their association with a range of outcomes (e.g., chronic health conditions, mental health, risk taking behaviours) with diverse results (Wang et al., 2024). Additional analysis of intergenerational ACEs, through means like latent class analysis, may further clarify the current proposed interpretation of this study’s findings. Research that considers the potential compounding and interacting nature of ACEs may be more realistic to parents’ experiences and better able to inform services. Service-providers are tasked with supporting the individual wholistically and in the context of multiple adversities and, thus, should be supported by research that does the same.

Compared to a representative Irish sample (Nixon, 2021), the participants in the current study reported higher scores of conflict with their child and this score was significantly, positively correlated to their cumulative ACE score. The study’s data was drawn from evaluation measures completed by parents at the start of engagement with a family service that provides a wide range of supports including therapeutic services and family interventions. Thus, the high perceived conflict score may reflect parents’ perception of familial challenges, including conflict, that predated or sparked their service engagement. The correlation between ACEs and conflict could indicate that ACEs impact parenting factors. ACEs are associated with increased interpersonal difficulties (e.g., cold/distant, socially inhibited) mediated by decreases in emotional regulation (Poole et al., 2018). Within the parenting relationship specifically, previous literature demonstrates a relationship between ACEs and challenges to parent-child attachment, emotional availability, and responsiveness (Narayan et al., 2021; Rowell & Neal-Barnett, 2021; Sroufe, 2017). The pathway between ACEs and challenges with parenting can be affected by factors specific to the adversity experienced. For example, previous literature poses that parents with CSA relive their trauma through their child (Roller, 2011; Wright et al., 2012), leading to over-protectiveness (Lind et al., 2018). This over-protectiveness may cause conflict, particularly as children age and seek greater autonomy (Arslan et al., 2023). Therefore, the conflict scores reported by parents here may reflect challenges to the parent-child relationship created or exasperated by parents’ adversity experiences.

When interpreting the relationship between ACEs and conflict, it is important to consider that the conflict measure captures parents’ perceptions of their relationship with their child, rather than an “objective” measure of conflict. This conceptualisation of the conflict measure could challenge the previously described potential nature of the relationship between ACEs and conflict. With this conceptualisation, the current results could reflect an association between ACEs and a perception of conflict. The demonstrated impact of ACEs on neurological and socioemotional development may lead to hyperarousal and hypersensitivity where cumulative ACEs score “prime” individuals to threat (Kendall-Tackett, 2000). Further, ACEs are associated with greater life stressors in adulthood including the higher reported quantity, severity, and effects of stressors (Mosley-Johnson et al., 2021) and, specifically, with stress related to parenting (Lange et al., 2019). The cumulation of hyperarousal and hypersensitivity and high stressors in daily life and parenting may affect how parents’ view and report their relationship with their child. Therefore, rather than reflecting greater conflict in the parent-child relationship, the findings could reflect a tendency for parents with ACEs to be more alert to conflict with their child.

These diverging interpretations carry distinct implications for clinical practice and the design of family support interventions. If the observed associations reflect a behavioural deficit in interactive regulation, interventions should prioritise psychoeducation and parent-training programmes (Sanders, 2012; Webster-Stratton & Reid, 2018), focused on non-reactive discipline and the modelling of warm, responsive interactions. Conversely, if the findings stem from a trauma-mediated perceptual bias, support should be rooted in trauma-informed care aimed at reducing parental hyperarousal and hypersensitivity. In such cases, clinical tools like video-feedback intervention (Juffer et al., 2008) could be particularly effective, as they provide an objective external perspective that helps parents recalibrate their internal representations (Berthelot et al., 2015), allowing them to distinguish between a child’s normative developmental challenges and perceived threats to authority or safety.

While reported conflict was positively correlated with cumulative ACEs, parents’ reported closeness with their child was negatively correlated with cumulative ACEs. However, closeness ratings by parents were at the higher end of the spectrum and similar to a representative Irish sample (Nixon, 2021). Research suggests that dynamics of high closeness and high conflict in parent-child relationships frequently coexist (e.g. Branje, 2018), but can be exacerbated in contexts where parents have a history of ACEs and may struggle, for example, with emotional regulation. This can lead to ambivalent or turbulent relationships characterised by intense emotional bonds alongside conflict, resentment, or mistrust. The findings from a qualitative study of parents with ACEs mirrored this result as participants described the cyclical nature of ACEs, challenges that ACEs present to parenting, and their desire to break this cycle through closeness with their child. The participants called for interventions that raised awareness of ACEs, built a supportive community, and provided parenting information to break the intergenerational cycle of ACEs (Woods-Jaeger et al., 2018). Collectively, this suggests a need to develop appropriate services to empower and resource parents with ACEs in minimising conflict while developing close relationships with their children.

The relationship between ACEs and outcomes is complex and affected by a wide range of moderating and mediating factors (e.g., perceived social support; McCutchen et al., 2023). The current finding may suggest that additional factors are relevant in the relationship between ACEs and parenting experiences. For example, in the present study, the initial association between parental history of child sexual abuse (CSA) and perceived parent-child closeness was attenuated upon the inclusion of socio-demographic variables and the total count of cumulative adverse childhood experiences (ACEs). This finding suggests that the impact of CSA on relational quality does not operate in isolation but is profoundly mediated by the current ecological context and the accumulation of multiple adversities. Socio-demographic factors, such as parental education, may serve as proxies for socio-economic stability and access to therapeutic resources, acting as protective mechanisms that foster parental resilience in the face of early trauma (Houtepen et al., 2018; Panisch et al., 2020). These results align with contemporary research highlighting how socio-economic security and social support can buffer the intergenerational transmission of trauma, enabling survivors of severe abuse to maintain strong affective bonds with their offspring, provided their current environment is stable and well-resourced (Templeton et al., 2025).

Furthermore, the significant influence of child age and sex on closeness levels reflects universal developmental and gender dynamics that may overshadow the distal effects of CSA. From a developmental perspective, child age is a primary determinant of relationship quality; as children transition into adolescence, there is a normative shift in attachment (with closeness as a dimension) characterised by a drive for autonomy, where emotional closeness with parents typically decreases as affiliation with peers increases. This process is frequently accompanied by heightened conflict, as adolescents challenge parental authority to define their own social roles and boundaries (Moretti & Peled, 2004; Smetana & Rote, 2019). Besides, gender dynamics significantly shape these relational patterns. Socialisation norms often encourage girls to be more expressive of affection and emotional vulnerability than boys, a trend reflected in the higher closeness scores reported for daughters in the present study (Borhan, 2024; Davis et al., 2020). These dynamics extend to the parents’ gendered approaches to child-rearing, where differing societal expectations for mothers and fathers regarding warmth and discipline can further alter the perceived bond (Fingerman et al., 2020). When these immediate, high-variance factors are accounted for, they effectively attenuate the statistical signal of the parent’s past CSA. As a distal factor occurring decades prior, the impact of CSA on current parenting is often ‘washed out’ by these proximal, everyday developmental shifts and gendered interactions that more directly dictate the current climate of the parent-child relationship. As a consequence, future research should explore this further with an aim to identify protective or buffering factors that can support parents who have had adversities in childhood. This may indicate meaningful places for interventions or targets for supportive services.

### Strengths, Limitations, and Future Directions

The current paper considered the co-occurring nature of ACEs to better understand the relationship between CSA specifically, and ACEs broadly, to parents’ perceptions of their relationship with their children. Within the analysis, a wide range of demographic variables were considered to create more complex models that led to different understandings of the relationships between these variables. Conducting this work in an Irish context with families who are accessing support services has the potential to support services in a context that is under-researched (McCutchen et al., 2022). Further, the results provide greater insight into a key population, namely those seeking supportive services who may have different concerns than the general public. Understanding this sample’s experience of ACEs and parent/child relationships can meaningfully advise services aimed at supporting similar families. Similarly, the inclusion of fathers fills a gap within ACE research that has frequently been conducted solely with mothers (Zhang et al., 2023).

Despite these strengths, the current study is limited by elements of the study design and population. The study utilised retrospective, self-report data which may raise concerns regarding recall bias. This has long been noted as a challenge within ACE research (Reuben et al., 2016) but may be the most practical and realistic way to record this data. The current data could have also been affected by a desirability bias as data was collected from parents in early sessions with a service-provider. Participants did report higher ACE scores and conflict compared to the general public (Nixon, 2021) indicating that there may be some comfort in sharing these experiences, but the possibility of this bias cannot be discounted.

The study’s findings and limitations suggest multiple directions for future research. As discussed above, a strength of this study was the inclusion of fathers, an understudied population in both parenting and ACE research (Deneault et al., 2025; Grafft et al., 2024). Future research should aim to include a greater portion of fathers and consider interacting gender dynamics (i.e., the gender of the parent and the child) in their modelling. Additionally, future research may benefit from incorporating data related to the parent-child relationship collected from children directly. This may add additional context and perspective to findings related to conflict and closeness by indicating how the child is experiencing these factors. Finally, future research should aim to further explore potential mediating factors in the relationship between ACEs and parenting experiences. A deeper understanding of factors that may support the perception of closeness, even in the presence of perceived conflict, may provide insight regarding specific targets for interventions for parents with ACEs.

In addition to implications for future research, the findings may support service-providers in supporting parents and children with ACEs. Specifically, the higher rate of ACEs reported by participants in the current study compared to the Irish general public and the association between ACEs and challenges in the family context indicate that family services may benefit from organisational approaches to addressing trauma. Trauma-informed care proposes that every organisational domain (e.g., financing, training, service-delivery) be designed based on an understanding of the prevalence and impact of trauma on the service-user population. By adapting this approach, organisations can align with key principles like safety, trust, and collaboration which may support service-users in sustaining service engagement (Substance Abuse and Mental Health Service Administration, 2014). Further, this approach calls for an understanding of the potential for staff to experience vicarious trauma as a result of their exposure to service-users’ experiences (McCann & Pearlman, 1990). Early reviews of trauma-informed care capture its use in mental health and child services and potential benefits to both service-users and providers (Fernández et al., 2023). A trauma-informed approach should be coherently integrated into services, considering the impact of ACEs on parents, their children, family dynamics, service-providers, and the organisation as a whole. Multiple guides and models have been developed to support organisations in adapting this approach (e.g., Substance Abuse and Mental Health Services Administration, 2023).

## Conclusions

The current study explores the relationship between cumulative ACEs, CSA as a specific experience, and perceptions of conflict and closeness in the parent-child relationship. Given the potential lifelong impact of ACEs and importance of the parenting relationship for both parents’ and children’s mental health, this study provides timely recommendations for both research and practice. To best support evidence-based practice, the current paper calls for a greater understanding of parents in the context of all their interacting and compounding adverse experiences, in addition to further exploration of protective factors. Through creating more in-depth models of ACEs and parenting relationships, evidence-based services can better support parents and promote healthy parent-child relationships.

## Data Availability

The authors do not have permission to share data.

## References

[CR1] Alexander, P. C., Teti, L., & Anderson, C. L. (2000). Childhood sexual abuse history and role reversal in parenting. *Child abuse & neglect,**24*(6), 829–838. 10.1016/s0145-2134(00)00142-3 10888021

[CR2] Amado, B. G., Arce, R., & Herraiz, A. (2015). Psychological injury in victims of child sexual abuse: A meta-analytic review. *Psychosocial Intervention,**24*(1), 49–62. 10.1016/j.psi.2015.03.002

[CR3] Arslan, İB., Lucassen, N., Keijsers, L., & Stevens, G. W. J. M. (2023). When too much help is of no help: Mothers’ and fathers’ perceived overprotective behavior and (mal)adaptive functioning in adolescents. *Journal of Youth and Adolescence,**52*(5), 1010–1023. 10.1007/s10964-022-01723-0 36633796 PMC10027782

[CR4] Barrett, B. (2009). The impact of childhood sexual abuse and other forms of childhood adversity on adulthood parenting. *Journal of Child Sexual Abuse,**18*(5), 489–512. 10.1080/10538710903182628 20183414

[CR5] Berthelot, N., Ensink, K., Bernazzani, O., Normandin, L., Luyten, P., & Fonagy, P. (2015). Intergenerational transmission of attachment in abused and neglected mothers: The role of trauma-specific reflective functioning. *Infant Mental Health Journal*, *36*(2), 200–212. 10.1002/imhj.21499 25694333

[CR6] Borhan, N. (2024). A review study about gender differences in expressive language: Spoken and written language differences. *Gelişim Ve Psikoloji Dergisi,**4*(8), 76–95. 10.51503/gpd.1231592

[CR7] Branje, S. (2018). Development of parent–adolescent relationships: Conflict interactions as a mechanism of change. *Child Development Perspectives,**12*, 171–176. 10.1111/cdep.12278

[CR8] Calatrava, M., Swords, L., & Spratt, T. (2023). Socio-emotional adjustment in children attending family centres: The role of the parent–child relationship. *The British Journal of Social Work,**53*(5), 2725–2741. 10.1093/bjsw/bcac241

[CR999] Davis, M. M., Modi, H. H., & Rudolph, K. D. (2020). Sex, Gender, and Emotion. In S. Hupp, & J. D. Jewell (Eds.), *The Encyclopedia of Child and Adolescent Development* (pp. 1–12). John Wiley & Sons, Inc. 10.1002/9781119171492.wecad179

[CR9] Deblinger, E., & Heflin, A. H. (1996). *Treating sexually abused children and their nonoffending parents: a cognitive behavioral approach*. Sage Publications.

[CR10] Deneault, A., Laquerre, D., Beaulieu, L., Myre, G., Racine, N., & Madigan, S. (2025). Prevalence and correlates of adverse childhood experiences in fathers: A systematic review and meta-analysis. *Child Abuse & Neglect*. 10.1016/j.chiabu.2025.107651 40961818

[CR11] DiLillo, D., & Damashek, A. (2003). Parenting characteristics of women reporting a history of childhood sexual abuse. *Child Maltreatment,**8*(4), 319–333. 10.1177/1077559503257104 14604178

[CR12] Easton, S. D. (2012). Understanding adverse childhood experiences (ACE) and their relationship to adult stress among male survivors of childhood sexual abuse. *Journal of Prevention & Intervention in the Community,**40*(4), 291–303. 10.1080/10852352.2012.707446 22970782

[CR13] ECPAT International (2016). *Terminology guidelines for the protection of children from sexual exploitation and sexual abuse.* https://goo.su/nTlL

[CR14] Felitti, V. J., Anda, R. F., Nordenberg, D., Williamson, D. F., Spitz, A. M., Edwards, V., Koss, M. P., & Marks, J. S. (1998). Relationship of childhood abuse and household dysfunction to many of the leading causes of death in adults. The Adverse Childhood Experiences (ACE) Study. *American Journal of Preventive Medicine,**14*(4), 245–258. 10.1016/s0749-3797(98)00017-8 9635069

[CR15] Fergusson, D. M., McLeod, G. F., & Horwood, L. J. (2013). Childhood sexual abuse and adult developmental outcomes: Findings from a 30-year longitudinal study in New Zealand. *Child Abuse & Neglect,**37*(9), 664–674. 10.1016/j.chiabu.2013.03.013 23623446

[CR16] Fernández, V., Gausereide-Corral, M., Valiente, C., & Sánchez-Iglesias, I. (2023). Effectiveness of trauma-informed care interventions at the organizational level: A systematic review. *Psychological Services*, *20*(4), 849–862. 10.1037/ser0000737 36689374

[CR17] Fingerman, K. L., Huo, M., & Birditt, K. S. (2020). Mothers, fathers, daughters, and sons: Gender differences in adults’ intergenerational ties. *Journal of Family Issues,**41*(9), 1597–1625. 10.1177/0192513X19894369 38239383 PMC10795962

[CR18] Finkelhor, D., & Browne, A. (1985). The traumatic impact of child sexual abuse: A conceptualization. *The American Journal of Orthopsychiatry,**55*(4), 530–541. 10.1111/j.1939-0025.1985.tb02703.x 4073225

[CR19] Grafft, N., Lo, B., Easton, S. D., Pineros-Leano, M., & Davison, K. K. (2024). Maternal and paternal adverse childhood experiences (ACEs) and offspring health and wellbeing: A scoping review. *Maternal and Child Health Journal,**28*(1), 52–66. 10.1007/s10995-023-03825-y 37914980

[CR20] Greene, C. A., Haisley, L., Wallace, C., & Ford, J. D. (2020). Intergenerational effects of childhood maltreatment: A systematic review of the parenting practices of adult survivors of childhood abuse, neglect, and violence. *Clinical Psychology Review*. 10.1016/j.cpr.2020.101891 PMC7476782 32745835

[CR21] Houtepen, L. C., Heron, J., Suderman, M. J., Fraser, A., & Howe, L. D. (2018). Association of adverse childhood experiences with educational attainment and adolescent health, and the role of socioeconomic factors: Analysis of a prospective cohort study. *The Lancet,**392*, S43. 10.1016/S0140-6736(18)32067-1 PMC7051040 32119668

[CR22] Hughes, K., Bellis, M. A., Hardcastle, K. A., Sethi, D., Butchart, A., Mikton, C., Jones, L., & Dunne, M. P. (2017). The effect of multiple adverse childhood experiences on health: A systematic review and meta-analysis. *The Lancet Public health,**2*(8), 356–366. 10.1016/S2468-2667(17)30118-4 29253477

[CR23] Hugill, M., Berry, K., & Fletcher, I. (2017). The association between historical childhood sexual abuse and later parenting stress: A systematic review. *Archives of Women’s Mental Health,**20*(2), 257–271. 10.1007/s00737-016-0708-3 28054214 PMC5344942

[CR24] Irish, L., Kobayashi, I., & Delahanty, D. L. (2010). Long-term physical health consequences of childhood sexual abuse: A meta-analytic review. *Journal of Pediatric Psychology,**35*(5), 450–461. 10.1093/jpepsy/jsp118 20022919 PMC2910944

[CR25] Juffer, F., Bakermans-Kranenburg, M. J., & van IJzendoorn, M. H. (Eds.). (2008). *Promoting positive parenting: An attachment-based intervention*. Taylor & Francis Group/Lawrence Erlbaum Associates.

[CR26] Kendall-Tackett, K. A. (2000). Physiological correlates of childhood abuse: Chronic hyperarousal in PTSD, depression, and irritable bowel syndrome. *Child Abuse & Neglect,**24*(6), 799–810. 10.1016/s0145-2134(00)00136-8 10888019

[CR27] Lacey, R. E., & Minnis, H. (2019). Practitioner review: Twenty years of research with adverse childhood experience scores - Advantages, disadvantages and applications to practice. *Journal of Child Psychology and Psychiatry,**61*(2), 116–130. 10.1111/jcpp.13135 31609471

[CR28] Lacey, R. E., Howe, L. D., Kelly-Irving, M., Bartley, M., & Kelly, Y. (2020). The clustering of adverse childhood experiences in the Avon Longitudinal Study of Parents and Children: Are gender and poverty important? *Journal of Interpersonal Violence,**37*(5–6), 2218–2241. 10.1177/0886260520935096 32639853 PMC8918866

[CR29] Lange, B. C. L., Callinan, L. S., & Smith, M. V. (2019). Adverse childhood experiences and their relation to parenting stress and parenting practices. *Community Mental Health Journal,**55*(4), 651–662. 10.1007/s10597-018-0331-z 30194589 PMC6447511

[CR30] Lind, M. J., Brown, R. C., Sheerin, C. M., York, T. P., Myers, J. M., Kendler, K. S., & Amstadter, A. B. (2018). Does parenting influence the enduring impact of severe childhood sexual abuse on psychiatric resilience in adulthood? *Child Psychiatry & Human Development,**49*(1), 33–41. 10.1007/s10578-017-0727-y 28488144 PMC5680128

[CR31] MacIntosh, H. B., & Ménard, A. D. (2021). Couple and parenting functioning of childhood sexual abuse survivors: A systematic review of the literature (2001–2018). *Journal of Child Sexual Abuse*, *30*(3), 353–384. 10.1080/10538712.2020.1847227 33491586

[CR32] Madigan, S., Deneault, A. A., Racine, N., Park, J., Thiemann, R., Zhu, J., Dimitropoulos, G., Williamson, T., Fearon, P., Cénat, J. M., McDonald, S., Devereux, C., & Neville, R. D. (2023). Adverse childhood experiences: A meta-analysis of prevalence and moderators among half a million adults in 206 studies. *World psychiatry: Official journal of the World Psychiatric Association (WPA),**22*(3), 463–471. 10.1002/wps.21122 37713544 PMC10503911

[CR33] McCann, I. L., & Pearlman, L. A. (1990). Vicarious traumatization: A framework for understanding the psychological effects of working with victims. *Journal of traumatic stress*, *3*(1), 131–149.

[CR34] McCutchen, C., Hyland, P., Shevlin, M., & Cloitre, M. (2022). The occurrence and co-occurrence of ACEs and their relationship to mental health in the United States and Ireland. *Child Abuse & Neglect*. 10.1016/j.chiabu.2022.105681 35643057

[CR35] McCutchen, C., Hyland, P., Maercker, A., Thoma, M. V., & Rohner, S. L. (2023). The effects of social support on ACEs and mental health in Ireland. *Journal of Loss and Trauma*, *28*(4), 377–388. 10.1080/15325024.2022.2124264

[CR36] Monson, T., Swords, L., & Spratt, T. (2022). Establishing outcome measures in practice: Developing a model for services working therapeutically with children and families. *The British Journal of Social Work*, *52*(6), 3501–3521. 10.1093/bjsw/bcab259

[CR37] Moretti, M. M., & Peled, M. (2004). Adolescent-parent attachment: Bonds that support healthy development. *Paediatrics & child health*, *9*(8), 551–555. 10.1093/pch/9.8.551 19680483 PMC2724162

[CR38] Mosley-Johnson, E., Campbell, J. A., Garacci, E., Walker, R. J., & Egede, L. E. (2021). Stress that Endures: Influence of Adverse Childhood Experiences on Daily Life Stress and Physical Health in Adulthood. *Journal of affective disorders*, *284*, 38–43. 10.1016/j.jad.2021.02.018 33582431 PMC8040325

[CR39] Narayan, A. J., Lieberman, A. F., & Masten, A. S. (2021). Intergenerational transmission and prevention of adverse childhood experiences (ACEs). *Clinical Psychology Review,**85*, Article 101997. 10.1016/j.cpr.2021.101997 33689982

[CR40] Nixon, E. (2021). *Social*-emotional and behavioural outcomes in early adolescence. government publications.

[CR41] Nixon, E., & Swords, L. (2016). Is Family Structure a Source of Inequality in Children’s Lives? In J. Williams, E. Nixon, E. Smyth, & D. Watson (Eds.), *Cherishing all the children equally? Ireland 100 years on from the Easter Rising* (pp. 58–79). Oak Tree Press.

[CR42] Nixon, E., Swords, L., & Murray, A. (2013). Growing up in Ireland: parenting and infant development. *Dublin: Department Of Children And Youth Affairs*.

[CR43] Panisch, L. S., LaBrenz, C. A., Lawson, J., Gerlach, B., Tennant, P. S., Nulu, S., & Faulkner, M. (2020). Relationships between adverse childhood experiences and protective factors among parents at-risk for child maltreatment. *Children and Youth Services Review,**110*, Article 104816. 10.1016/j.childyouth.2020.104816

[CR44] Pastor-Cerezo, V., & Iborra Cuéllar, A. (2026a). From trauma to bonding: the role of attachment in the relationship between child sexual abuse and adult intimacy. *Journal of Child & Adolescent Trauma*. 10.1007/s40653-026-00845-y

[CR45] Pastor-Cerezo, V., & Iborra Cuéllar, A. (2026b). The echo of trauma: An interpretative phenomenological analysis of the impact of childhood sexual abuse on adult attachment and intimacy. *European Journal of Trauma & Dissociation = Revue Europâeenne Du Trauma Et De La Dissociation,**10*(1), Article 100652. 10.1016/j.ejtd.2026.100652

[CR46] Pianta, R. C. (1992). Child-parent relationship scale. *Unpublished measure, University of Virginia*, *427*.

[CR47] Poole, J. C., Dobson, K. S., & Pusch, D. (2018). Do adverse childhood experiences predict adult interpersonal difficulties? The role of emotion dysregulation. *Child Abuse & Neglect,**80*, 123–133. 10.1016/j.chiabu.2018.03.006 29604503

[CR48] Ports, K. A., Ford, D. C., & Merrick, M. T. (2016). Adverse childhood experiences and sexual victimization in adulthood. *Child Abuse & Neglect,**51*, 313–22. 10.1016/j.chiabu.2015.08.017 26386753 PMC4713310

[CR49] Reuben, A., Moffitt, T. E., Caspi, A., Belsky, D. W., Harrington, H., Schroeder, F., Hogan, S., Ramrakha, S., Poulton, R., & Danese, A. (2016). Lest we forget: Comparing retrospective and prospective assessments of adverse childhood experiences in the prediction of adult health. *Journal of Child Psychology and Psychiatry and Allied Disciplines,**57*(10), 1103–1112. 10.1111/jcpp.12621 27647050 PMC5234278

[CR50] Roller, C. G. (2011). Moving beyond the pain: Women’s responses to the perinatal period after childhood sexual abuse. *Journal of Midwifery & Women’s Health,**56*(5), 488–493. 10.1111/j.1542-2011.2011.00051.x 23181647

[CR51] Rowell, T., & Neal-Barnett, A. (2021). A systematic review of the effect of parental adverse childhood experiences on parenting and child psychopathology. *Journal of child & adolescent trauma,**15*(1), 167–180. 10.1007/s40653-021-00400-x 35222782 PMC8837768

[CR52] Sanders, M. R. (2012). Development, evaluation, and multinational dissemination of the Triple P-Positive Parenting Program. *Annual review of clinical psychology,**8*, 345–379. 10.1146/annurev-clinpsy-032511-143104 22149480

[CR53] Schickedanz, A., Escarce, J. J., Halfon, N., Sastry, N., & Chung, P. J. (2021). Intergenerational associations between parents’ and children’s adverse childhood experience scores. *Children*, *8*(9), 747. 10.3390/children8090747 34572179 PMC8466272

[CR54] Smetana, J. G., & Rote, W. M. (2019). Adolescent-parent relationships: Progress, processes, and prospects. *Annual Review of Developmental Psychology,**1*(1), 41–68. 10.1146/annurev-devpsych-121318-084903

[CR55] Spratt, T., Swords, L., & Vilda, D. (2021). Outcomes for families referred to family centres: Using validated instruments to chart changes in psychological functioning, relationships and children’s coping strategies over time. *The British Journal of Social Work,**51*(3), 794–815. 10.1093/bjsw/bcaa222

[CR56] Sroufe, L. A. (2017). The place of attachment in development. In J. Cassidy, & P. R. Shaver (Eds.), *The Handbook of Attachment* (3rd ed.). Guildford.

[CR57] Substance Abuse and Mental Health Services Administration (2023). *P*r*actical Guide for Implementing a Trauma-Informed Approach.* SAMHSA Publication No. PEP23-06-05-005. Rockville, MD: National Mental Health and Substance Use Policy Laboratory. Substance Abuse and Mental Health Services Administration.

[CR58] Substance Abuse and Mental Health Services Administration. (2014). *SAMHSA’s concept of trauma and guidance for a trauma-informed approach. HHS Publication No. (SMA) 14-4884*. Substance abuse and mental health services administration.

[CR59] Swedo, E. A., Aslam, M. V., Dahlberg, L. L., Niolon, P. H., Guinn, A. S., Simon, T. R., & Mercy, J. A. (2023). Prevalence of adverse childhood experiences among U.S. adults - Behavioral Risk Factor Surveillance System, 2011–2020. *MMWR. Morbidity and mortality weekly report,**72*(26), 707–715. 10.15585/mmwr.mm7226a2 37384554 PMC10328489

[CR60] Swords, L., Kennedy, M., & Spratt, T. (2024). Pathways explaining the intergenerational effects of ACEs: The mediating roles of mothers’ mental health and the quality of their relationships with their children. *Journal of Applied Developmental Psychology*. 10.1016/j.appdev.2024.101644

[CR61] Templeton, J. M., Dixon, W. E., Jr., Williams, S., Morelen, D., Driggers-Jones, L., & Robertson, C. (2025). The mediating role of social support on the link between adverse childhood experiences and adult mental health. *Journal of experimental child psychology,**252*, Article 106148. 10.1016/j.jecp.2024.106148 39706049

[CR62] UNICEF, United Nations Children’s Fund. (2023). *International Classification of Violence against Children*. UNICEF.

[CR63] Wang, X., Jiang, L., Barry, L., Zhang, X., Vasilenko, S. A., & Heath, R. D. (2024). A scoping review on adverse childhood experiences studies using latent class analysis: Strengths and challenges. *Trauma Violence & Abuse*, *25*(2), 1695–1708. 10.1177/15248380231192922 37594222

[CR64] Webster-Stratton, C., & Reid, M. J. (2018). The Incredible Years parents, teachers, and children training series: A multifaceted treatment approach for young children with conduct problems. In J. R. Weisz, & A. E. Kazdin (Eds.), *Evidence-based psychotherapies for children and adolescents* (3rd ed., pp. 122–141). The Guilford Press.

[CR65] Woods-Jaeger, B. A., Cho, B., Sexton, C. C., Slagel, L., & Goggin, K. (2018). Promoting resilience: Breaking the intergenerational cycle of adverse childhood experiences. *Health Education & Behavior*, *45*(5), 772–780. 10.1177/1090198117752785 29433342

[CR66] Wright, M. O., Fopma-Loy, J., & Oberle, K. (2012). In their own words: The experience of mothering as a survivor of childhood sexual abuse. *Development and Psychopathology*, *24*(2), 537–552. 10.1017/S0954579412000144 22559129

[CR67] Zhang, L., Mersky, J. P., Gruber, A. M. H., & Kim, J. (2023). Intergenerational transmission of parental adverse childhood experiences and children’s outcomes: A scoping review. *Trauma Violence & Abuse*, *24*(5), 3251–3264. 10.1177/15248380221126186 36205317

